# Predicting cognitive impairment in Parkinson’s disease: a machine learning approach based on clinical and neuropsychological data

**DOI:** 10.3389/fneur.2025.1709386

**Published:** 2025-12-19

**Authors:** Meili Yang, Chuxin Wang, Jinying Zhang, Yao Xiao, Yafang Chen, Zeming Guo, Jiayin Wang, Jinzhong Huang

**Affiliations:** 1 Department of Geriatrics, The Second Affiliated Hospital of Fujian Medical University, Quanzhou, China; 2 Fujian Medical University Union Hospital, Fuzhou, China; 3 Department of Neurosurgery, The Second Affiliated Hospital of Fujian Medical University, Quanzhou, China

**Keywords:** Parkinson’s disease, cognitive impairment, prediction, machine learning, Random Forest, outpatient management

## Abstract

**Background:**

Cognitive impairment is a common and disabling non-motor symptom of Parkinson’s disease, markedly diminishing quality of life and elevating caregiver burden. Although considerable research has been conducted, the early prediction of cognitive impairment remains challenging owing to heterogeneous clinical presentations, variations in treatment adherence, and the inherent limitations in sensitivity of conventional biomarkers and cognitive assessment tools.

**Methods and materials:**

A retrospective cohort study involving 514 Parkinson’s disease patients who had complete baseline data and a minimum of 6 months of follow-up. Participants were randomly allocated into a training cohort (*n* = 359) and a test cohort (*n* = 155). Demographic, clinical, biochemical, and neuropsychological variables were obtained at baseline. Cognitive impairment was defined based on Mini-Mental State Examination scores falling below education-adjusted thresholds and further validated using the Montreal Cognitive Assessment. Multiple machine learning models—including Random Forest, Logistic Regression, Gradient Boosting, CatBoost, and Support Vector Machine—were developed and evaluated using the area under the receiver operating characteristic curve, accuracy, recall, F1-score, calibration, and decision curve analysis. Feature importance analysis was performed to identify key predictive variables.

**Results:**

During follow-up, patients who developed cognitive impairment were significantly older and had longer disease duration, lower levels of albumin, hematocrit, and blood lipids, as well as a higher prevalence of hypertension. Feature selection identified: Age, Platelet count, Time from diagnosis to baseline visit, Apolipoprotein B, and Hematocrit as the predictors. The Random Forest model demonstrated the best overall performance, with the area under the receiver operating characteristic curve = 0.846, accuracy = 0.75, and an F1-score = 0.775, followed by CatBoost and Logistic Regression. Calibration and decision curve analyses confirmed stable probability estimation and superior clinical utility of Random Forest compared with “treat all” or “treat none” strategies. Further use the Montreal Cognitive Assessment score to verify the stability of the model.

**Conclusion:**

Machine learning models integrating multimodal clinical and neuropsychological data demonstrate high accuracy in predicting cognitive impairment in Parkinson’s disease, with Random Forest emerging as the most reliable approach. This framework provides a practical tool for early risk stratification, potentially enabling timely interventions and individualized management to reduce the burden of cognitive decline in Parkinson’s disease.

## Introduction

Parkinson’s disease (PD) is a common neurodegenerative disorder marked not only by classical motor symptoms but also by a wide spectrum of non-motor manifestations, among which cognitive impairment and dementia are particularly disabling. Epidemiological studies have shown that cognitive decline occurs in a substantial proportion of PD patients, with prevalence estimates ranging from 20% to over 40% depending on the diagnostic criteria applied, and disease severity identified as a key predictor of dementia risk (1). Longitudinal cohort investigations further revealed that distinct cognitive syndromes exist in PD, and that genetic and clinical markers, such as the MAPT H1/H1 haplotype and specific baseline cognitive deficits, are strong predictors of progression to dementia (2). Importantly, cognitive decline and neuropsychiatric symptoms such as depression, anxiety, and apathy significantly worsen patients’ quality of life and are closely associated with caregiver burden, often surpassing the impact of motor disability (3). Indeed, longitudinal analyses demonstrate that deterioration in quality of life is most strongly related to worsening cognitive and psychosocial domains, rather than motor symptoms alone (4). Parallel studies confirm that both motor and non-motor features, including stigma and emotional well-being, directly influence caregivers’ stress levels and overall burden (5). In advanced PD, caregiver burden is further intensified, being linked to patient quality of life, speech difficulties, and cognitive deterioration, with therapeutic strategies such as continuous dopaminergic delivery systems offering partial relief (6). Taken together, these findings underscore that cognitive impairment represents not only a common and predictable outcome of PD but also a critical factor diminishing patients’ quality of life and increasing caregiver burden—highlighting the pressing need for early detection and the development of robust predictive models.

Although PD patients often require long-term management, the majority are treated in outpatient settings, where the variability in visit timing, location, and examination protocols contributes to heterogeneous clinical data and inconsistent follow-up. As a result, treatment adherence varies widely among patients, leading to significant differences in disease progression and outcomes. Early identification of patients at risk for cognitive impairment (CI) remains a major challenge. Against this backdrop, there is a clear demand for predictive models that not only incorporate multimodal clinical information but also effectively capture the heterogeneity of real-world outpatient care. Many prior investigations have hinged on the use of specialized, resource-intensive biomarkers, such as neuroimaging and cerebrospinal fluid measures (7). Similarly, cognitive assessment tools such as the Mini Mental Parkinson and SCOPA-COG scales demonstrate utility in rating deterioration, yet their sensitivity to early changes is insufficient for robust prognostication (8). Large cohort studies have further emphasized that baseline demographic and clinical parameters explain only part of the variability in cognitive outcomes, underscoring the need for more reliable predictive measures (9). In addition, although uric acid has been suggested as a potential neuroprotective factor, longitudinal data show inconsistent associations with cognitive decline, reflecting the complexity of disease mechanisms (10). These tools, however, may not be routinely available or scalable in real-world, heterogeneous clinical settings where data can often be fragmented or lacking, representing a large translational gap between the research environment and actual clinical practice. In sharp contrast to these approaches, the present study is uniquely designed to develop and validate a comprehensive and yet operationally practical predictive framework based on widely accessible clinical and neuropsychological parameters. By effectively harnessing the power of machine learning (ML) to integrate heterogeneous data sources, that can potentially overcome the challenges posed by fragmented outpatient data and improve prognostic accuracy without depending on specialized or resource-intensive biomarkers. Ultimately, this work holds the promise to enable earlier interventions, optimize individualized treatment strategies, and alleviate the overall burden of cognitive decline on both patients and caregivers.

The principal contributions of this work are summarized as follows:

1. Development of a practical predictive framework: The study developed and validated multiple ML models to predict cognitive impairment in Parkinson’s disease using exclusively routine clinical and neuropsychological data, thereby creating a tool readily applicable in heterogeneous outpatient settings.
2. Identification of accessible predictors: Through robust feature importance analysis, the study identified key predictive variables such as Age, Platelet count, Time from diagnosis to baseline visit, Apolipoprotein B, and Hematocrit, demonstrating the significant prognostic value embedded in commonly available clinical data.
3. Determination of an optimal model: Comprehensive evaluation of the research established the Random Forest algorithm as the most robust and clinically useful model, highlighting its suitability for handling complex, non-linear relationships in real-world patient data.
4. Rigorous and clinically relevant validation: The current analysis ensured the robustness and generalizability of the findings by validating the model’s performance not only on an independent test set but also using an alternative cognitive assessment tool (MoCA), and by rigorously assessing clinical utility through decision curve analysis.
5. Bridging the gap between research and clinical practice: This study addresses the critical gap between research-focused biomarkers and everyday clinical needs by providing a data-driven framework that leverages existing outpatient data to facilitate early risk stratification and personalized management strategies.

## Methods and materials

### Patient enrollment

**Figure 1:**
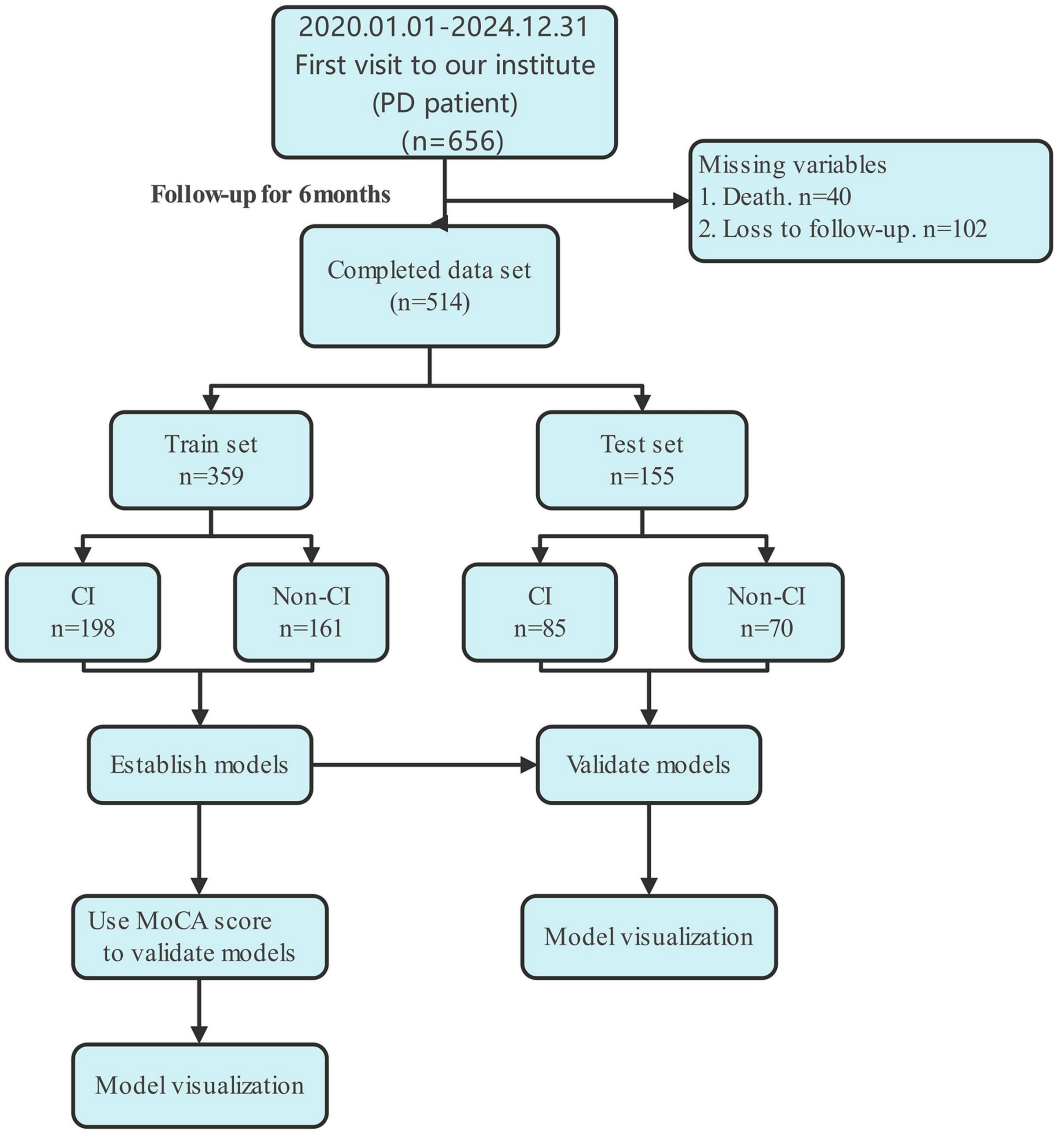
Flowchart of patient selection, model development, and validation.

Between January 1, 2020, and December 31, 2024, a total of 656 patients with PD attending their first visit at the Second Affiliated Hospital of Fujian Medical University were screened. After excluding 40 patients due to mortality and 102 lost to follow-up, 514 patients with complete datasets and at least 6 months of follow-up were included in the analysis. These participants were randomly allocated into a training set (*n* = 359, CI 198 vs. non-CI 161) and a test set (*n* = 155, CI 85 vs. Non-CI 70). Prediction models were developed using the training cohort and validated in the independent test cohort. Model performance was further verified using MoCA scores, and the results were visualized to enhance interpretability (Figure 1).

### The definition of CI

CI was primarily defined by the Mini-Mental State Examination (MMSE), with a score below the education-adjusted cutoff (typically <24) indicating CI. As an external validation, the MoCA was also applied, with a cutoff of <26 points used to define CI.

### Data collection

Demographic, clinical, and laboratory variables were collected at baseline through electronic medical records, including age, sex, time from diagnosis to baseline visit, comorbidities, hematological and biochemical indices (e.g., hematocrit, albumin, lipid profiles), and lifestyle factors. Cognitive function was assessed using standardized neuropsychological scales. All patients who included in the study met the inclusion criteria: (1) Age >18 years. (2) Patients diagnosed with PD at their first visit to the hospital. (3) Availability of complete demographic, clinical, and laboratory data at baseline. (4) No cognitive impairment at baseline, as determined by standardized neuropsychological assessment (MMSE and MoCA). (5) Patients with at least 6 months of follow-up after baseline evaluation. And other patients excluded from the study by the criteria: (1) Patients who died during the follow-up period. (2) Patients lost to follow-up or with incomplete clinical or neuropsychological data. (3) Patients with pre-existing CI at baseline.

### Follow-up

All patients were followed for a period of 6 months after the baseline assessment. Cognitive status was re-evaluated at the end of the follow-up period, and clinical outcomes were confirmed by trained neurologists who were blinded to the model predictions. The start of follow-up for each patient was defined as the time of their first visit to the hospital, and the end of follow-up was set at 6 months thereafter. The occurrence of CI was recorded as a positive case.

### Statistical analysis

Statistical analyses were performed using R software (version 4.3.2). Continuous variables were expressed as mean ± standard deviation and compared between groups using Student’s t-test or Mann–Whitney U test, depending on data distribution. Categorical variables were presented as frequencies and percentages and compared using the Chi-square test or Fisher’s exact test as appropriate. To construct prediction models for CI, multiple ML algorithms were applied, including Random Forest (RF), Logistic Regression (LR), Gradient Boosting, Support Vector Machine, and others. Model training was conducted on the training cohort, with hyper-parameter tuning performed via cross-validation. The independent test set was used for external validation. Model performance was evaluated using multiple metrics, including area under the receiver operating characteristic curve (AUROC), accuracy, recall, specificity, F1-score, and Matthews correlation coefficient (MCC). Calibration curves and decision curve analysis (DCA) were employed to assess calibration and clinical utility. Additionally, feature importance analysis was conducted to identify the most influential predictors. The primary predictive outcome was CI defined by the MMSE, while validation was further performed using CI defined by the MoCA scale. A two-sided *p*-value <0.05 was considered statistically significant.

## Results

### Patient demography

Baseline demographic and clinical characteristics are summarized in Table 1. Patients who developed CI were significantly older compared to those without CI in both the training and test cohorts (*p* < 0.001). Time from diagnosis to baseline visit was also longer in the CI group, highlighting “Time” as an important factor (*p* = 0.002 in the training set; *p* < 0.001 in the test set). Several laboratory indicators showed significant associations. Lower albumin levels were consistently observed in the CI group (*p* < 0.001 and *p* = 0.001, respectively), while the albumin-to-globulin ratio was reduced only in the test set (*p* = 0.007). Hematological parameters demonstrated differences as well, with reduced lymphocyte counts (*p* = 0.018), lower hematocrit (*p* < 0.001 and *p* = 0.022), and elevated hemoglobin A1c (HbA1c) in the test cohort (*p* = 0.032). Lipid metabolism markers revealed consistent trends, as patients with CI had lower total cholesterol (*p* = 0.005 in both sets), lower low-density lipoprotein (LDL-C) (*p* = 0.002 and *p* < 0.001), and reduced triglyceride, apolipoprotein A, and apolipoprotein B (APOB) levels (all *p* < 0.05). In addition, free triiodothyronine (FT3) was significantly lower in the CI group of the training set (*p* < 0.001). Regarding comorbidities, hypertension was more prevalent among CI patients in both cohorts (*p* = 0.047 and *p* < 0.001), and coronary artery disease was significantly enriched in the CI subgroup of the training set (*p* = 0.004). Interestingly, surgery history showed a difference only in the test set (*p* = 0.003), while other factors such as gender, diabetes, smoking, and drinking were not statistically different.

**Table 1:** Demographic characteristics of the training set and test set.

**Variable**	**Train Set**				**Test Set**			
	**Overall***N* = 359	**Non-CI***N* = 161	**CI***N* = 198	***P*-value**	**Overall***N* = 155	**Non-CI***N* = 70	**CI***N* = 85	***P*-value**
Age, mean ± sd	68.77 ± 9.69	63.04 ± 9.12	73.42 ± 7.37	<0.001	68.39 ± 10.35	61.86 ± 9.75	73.78 ± 7.31	<0.001
Gender, *n* (p%)				0.208				0.123
Female	176.00 (49.03%)	73.00 (45.34%)	103.00 (52.02%)		78.00 (50.32%)	40.00 (57.14%)	38.00 (44.71%)	
Male	183.00 (50.97%)	88.00 (54.66%)	95.00 (47.98%)		77.00 (49.68%)	30.00 (42.86%)	47.00 (55.29%)	
Time, mean ± sd	39.07 ± 33.88	32.94 ± 30.45	44.06 ± 35.74	0.002	37.93 ± 33.01	24.36 ± 25.96	49.11 ± 34.13	<0.001
ALB, mean ± sd	40.71 ± 4.55	41.66 ± 4.37	39.93 ± 4.55	<0.001	40.71 ± 4.78	42.04 ± 4.14	39.62 ± 5.02	0.001
GLB, mean ± sd	26.66 ± 4.90	26.32 ± 4.88	26.94 ± 4.92	0.236	26.91 ± 4.20	26.44 ± 4.13	27.29 ± 4.23	0.210
A/G, mean ± sd	1.63 ± 1.13	1.74 ± 1.64	1.53 ± 0.34	0.112	1.56 ± 0.33	1.63 ± 0.31	1.49 ± 0.33	0.007
WBC, mean ± sd	6.72 ± 1.82	6.81 ± 1.83	6.64 ± 1.81	0.376	6.76 ± 2.06	6.63 ± 1.87	6.86 ± 2.22	0.484
NEU, mean ± sd	4.47 ± 1.66	4.50 ± 1.67	4.44 ± 1.66	0.735	4.56 ± 1.94	4.42 ± 1.77	4.68 ± 2.07	0.404
LYM, mean ± sd	1.59 ± 0.54	1.67 ± 0.55	1.53 ± 0.53	0.018	1.58 ± 0.53	1.60 ± 0.48	1.57 ± 0.58	0.738
HCT, mean ± sd	0.39 ± 0.05	0.40 ± 0.05	0.38 ± 0.04	<0.001	0.39 ± 0.04	0.40 ± 0.05	0.38 ± 0.04	0.022
PLT, mean ± sd	221.84 ± 55.83	226.58 ± 57.92	217.98 ± 53.91	0.150	223.31 ± 63.28	230.47 ± 66.66	217.41 ± 60.10	0.207
FPG, mean ± sd	5.60 ± 1.23	5.63 ± 1.33	5.58 ± 1.16	0.699	5.72 ± 1.28	5.65 ± 1.19	5.77 ± 1.35	0.548
Hbc, mean ± sd	5.95 ± 0.74	5.96 ± 0.85	5.93 ± 0.64	0.745	6.01 ± 0.76	5.86 ± 0.66	6.12 ± 0.83	0.032
URIC, mean ± sd	311.84 ± 93.93	321.69 ± 88.16	303.83 ± 97.86	0.070	306.23 ± 98.51	300.27 ± 102.40	311.13 ± 95.52	0.499
CHOL, mean ± sd	4.27 ± 1.06	4.45 ± 1.09	4.13 ± 1.02	0.005	4.23 ± 1.07	4.50 ± 1.07	4.01 ± 1.03	0.005
HDL, mean ± sd	1.22 ± 0.31	1.26 ± 0.30	1.19 ± 0.31	0.047	1.26 ± 0.33	1.29 ± 0.28	1.24 ± 0.36	0.343
LDL, mean ± sd	2.61 ± 0.90	2.77 ± 0.90	2.47 ± 0.87	0.002	2.60 ± 0.93	2.88 ± 0.94	2.36 ± 0.87	<0.001
TG, mean ± sd	1.16 ± 0.50	1.24 ± 0.54	1.09 ± 0.46	0.008	1.11 ± 0.47	1.15 ± 0.45	1.07 ± 0.48	0.271
APOA, mean ± sd	1.24 ± 0.25	1.28 ± 0.23	1.20 ± 0.26	0.004	1.25 ± 0.28	1.31 ± 0.25	1.20 ± 0.30	0.013
APOB, mean ± sd	0.88 ± 0.26	0.92 ± 0.27	0.85 ± 0.25	0.010	0.86 ± 0.26	0.91 ± 0.27	0.82 ± 0.25	0.021
PLG, mean ± sd	3.17 ± 1.00	3.06 ± 0.98	3.26 ± 1.01	0.061	3.24 ± 1.00	3.12 ± 0.96	3.35 ± 1.02	0.146
D_Dimer, mean ± sd	2.36 ± 20.59	3.66 ± 30.66	1.31 ± 2.20	0.333	1.11 ± 2.17	0.62 ± 0.84	1.51 ± 2.77	0.006
HCY, mean ± sd	14.33 ± 9.59	14.16 ± 12.85	14.47 ± 5.74	0.781	15.34 ± 11.97	14.93 ± 15.98	15.68 ± 7.25	0.715
FT3, mean ± sd	4.36 ± 1.30	4.62 ± 1.50	4.14 ± 1.06	<0.001	4.35 ± 1.15	4.53 ± 0.88	4.20 ± 1.31	0.061
FT4, mean ± sd	16.80 ± 3.46	16.76 ± 3.60	16.83 ± 3.36	0.850	16.60 ± 2.77	16.95 ± 2.83	16.30 ± 2.70	0.149
TSH, mean ± sd	2.08 ± 1.99	2.02 ± 1.38	2.13 ± 2.38	0.562	2.00 ± 1.29	2.15 ± 1.39	1.89 ± 1.19	0.223
Education, *n* (p%)				0.092				0.383
Illiterate	12.00 (3.34%)	2.00 (1.24%)	10.00 (5.05%)		7.00 (4.52%)	2.00 (2.86%)	5.00 (5.88%)	
Primary school	37.00 (10.31%)	12.00 (7.45%)	25.00 (12.63%)		19.00 (12.26%)	6.00 (8.57%)	13.00 (15.29%)	
Junior high school	27.00 (7.52%)	13.00 (8.07%)	14.00 (7.07%)		9.00 (5.81%)	6.00 (8.57%)	3.00 (3.53%)	
High school	7.00 (1.95%)	4.00 (2.48%)	3.00 (1.52%)		4.00 (2.58%)	1.00 (1.43%)	3.00 (3.53%)	
College	10.00 (2.79%)	7.00 (4.35%)	3.00 (1.52%)		6.00 (3.87%)	2.00 (2.86%)	4.00 (4.71%)	
Other	266.00 (74.09%)	123.00 (76.40%)	143.00 (72.22%)		110.00 (70.97%)	53.00 (75.71%)	57.00 (67.06%)	
Surgery, *n* (p%)				0.205				0.003
No	337.00 (93.87%)	154.00 (95.65%)	183.00 (92.42%)		145.00 (93.55%)	70.00 (100.00%)	75.00 (88.24%)	
Yes	22.00 (6.13%)	7.00 (4.35%)	15.00 (7.58%)		10.00 (6.45%)	0.00 (0.00%)	10.00 (11.76%)	
TBI, *n* (p%)				0.261				0.890
No	354.00 (98.61%)	160.00 (99.38%)	194.00 (97.98%)		153.00 (98.71%)	69.00 (98.57%)	84.00 (98.82%)	
Yes	5.00 (1.39%)	1.00 (0.62%)	4.00 (2.02%)		2.00 (1.29%)	1.00 (1.43%)	1.00 (1.18%)	
CAD, *n* (p%)				0.004				0.570
No	317.00 (88.30%)	151.00 (93.79%)	166.00 (83.84%)		137.00 (88.39%)	63.00 (90.00%)	74.00 (87.06%)	
Yes	42.00 (11.70%)	10.00 (6.21%)	32.00 (16.16%)		18.00 (11.61%)	7.00 (10.00%)	11.00 (12.94%)	
Smoking, *n* (p%)				0.244				0.098
No	330.00 (91.92%)	145.00 (90.06%)	185.00 (93.43%)		145.00 (93.55%)	68.00 (97.14%)	77.00 (90.59%)	
Yes	29.00 (8.08%)	16.00 (9.94%)	13.00 (6.57%)		10.00 (6.45%)	2.00 (2.86%)	8.00 (9.41%)	
Hpertension, *n* (p%)				0.047				<0.001
No	182.00 (50.70%)	91.00 (56.52%)	91.00 (45.96%)		97.00 (62.58%)	56.00 (80.00%)	41.00 (48.24%)	
Yes	177.00 (49.30%)	70.00 (43.48%)	107.00 (54.04%)		58.00 (37.42%)	14.00 (20.00%)	44.00 (51.76%)	
Diabetes, *n* (p%)				0.990				0.970
No	292.00 (81.34%)	131.00 (81.37%)	161.00 (81.31%)		122.00 (78.71%)	55.00 (78.57%)	67.00 (78.82%)	
Yes	67.00 (18.66%)	30.00 (18.63%)	37.00 (18.69%)		33.00 (21.29%)	15.00 (21.43%)	18.00 (21.18%)	
Drinking, *n* (p%)				0.511				0.808
No	348.00 (96.94%)	155.00 (96.27%)	193.00 (97.47%)		149.00 (96.13%)	67.00 (95.71%)	82.00 (96.47%)	
Yes	11.00 (3.06%)	6.00 (3.73%)	5.00 (2.53%)		6.00 (3.87%)	3.00 (4.29%)	3.00 (3.53%)	

CI, Cognitive impairment; Time, Time between diagnosis and initial presentation to our hospital (Month); ALB, Albumin (g/L); GLB, Globulin (g/L); A/G, Albumin-to-Globulin ratio; WBC, White Blood Cell (10_9_/L); NEU, Neutrophil (10_9_/L); LYM, Lymphocyte (10_9_/L); HCT, Hematocrit (L/L); PLT, Platelet (10_9_/L); HbA1c, Hemoglobin A1c (mmol/mol); URIC, Uric Acid (umol/L); FPG, Fasting Plasma Glucose (mmol/L); CHOL, Total Cholesterol (mmol/L); HDL, High-Density Lipoprotein (mmol/L); LDL, Low-Density Lipoprotein (mmol/L); TG, Triglyceride (mmol/L); APOA, Apolipoprotein A (g/L); APOB, Apolipoprotein B (g/L); PLG, Plasminogen (g/L); HCY, Homocysteine (umol/L); FT3, Free Triiodothyronine (pg/mL); FT4, Free Thyroxine (ng/dL); TSH, Thyroid-Stimulating Hormone (mIU/dL).

### Construct the predicted model by ML

The Table 2 showed that the decision tree-based feature selection method effectively identified the most predictive variables, with Age demonstrating the highest importance (0.23 ± 0.02), followed by platelet (PLT), Time (between diagnosis and initial presentation to the hospital), APOB and Hematocrit (HCT). This approach highlights the model’s ability to prioritize clinically relevant features while filtering out non-informative predictors, enhancing interpretability and reducing overfitting.

**Table 2:** Clinical prediction of optimized machine learning framework.

Features	Importances_mean	Importances_std
Age	0.23	0.02
PLT	0.03	0.02
Time	0.03	0.02
APOB	0.02	0.01
HCT	0.01	0.01
HCY	0.0	0.0
D_Dimer	0.0	0.0
PLG	0.0	0.0
FT3	0.0	0.0
FT4	0.0	0.0
TG	0.0	0.0
LDL	0.0	0.0
HDL	0.0	0.0
CHOL	0.0	0.0
URIC	0.0	0.0
Hbc	0.0	0.0
FPG	0.0	0.0
APOA	0.0	0.0
Gender	0.0	0.0
LYM	0.0	0.0
NEU	0.0	0.0
WBC	0.0	0.0
A_G	0.0	0.0
GLB	0.0	0.0
ALB	0.0	0.0
Drinking	0.0	0.0
CAD	0.0	0.0
Diabetes	0.0	0.0
Hpertension	0.0	0.0
Smoking	0.0	0.0
TBI	0.0	0.0
Surgery	0.0	0.0
Education	0.0	0.0
TSH	0.0	0.0

Time, Time from diagnosis to baseline visit (Month); ALB, Albumin (g/L); GLB, Globulin (g/L); A/G, Albumin-to-Globulin ratio; WBC, White blood cell (10^9^/L); NEU, Neutrophil (10^9^/L); LYM, Lymphocyte (10^9^/L); HCT, Hematocrit (L/L); PLT, Platelet (10^9^/L); HbA1c, Hemoglobin A1c (mmol/mol); URIC, Uric acid (umol/L); FPG, Fasting plasma glucose (mmol/L); CHOL, Total cholesterol (mmol/L); HDL, High-density lipoprotein (mmol/L); LDL, Low-density lipoprotein (mmol/L); TG, Triglyceride (mmol/L); APOA, Apolipoprotein A (g/L); APOB, Apolipoprotein B (g/L); PLG, Plasminogen (g/L); HCY, homocysteine (umol/L); FT3, Free triiodothyronine (pg/mL); FT4, Free thyroxine (ng/dL); TSH, Thyroid-stimulating hormone (mIU/dL).

In the optimized ML framework, feature importance analysis revealed that age was the most influential predictor of cognitive impairment in patients with PD, contributing substantially more than any other variable (mean importance = 0.23 ± 0.02). Other features with relatively modest but notable predictive value included PLT (0.03 ± 0.02), Time from diagnosis to baseline visit (Time, 0.03 ± 0.02), APOB (0.02 ± 0.01), and HCT (0.01 ± 0.01). By contrast, a large number of clinical and biochemical parameters—such as homocysteine, D-dimer, lipid profiles, thyroid hormones, and comorbidity indicators—showed negligible or no contribution, with mean importance values approaching zero. These findings suggest that the predictive performance of the optimized framework was primarily driven by a limited set of variables, with age emerging as the dominant determinant of model output, while most other clinical and laboratory features offered minimal incremental value.

### Model discrimination and calibration

**Figure 2:**
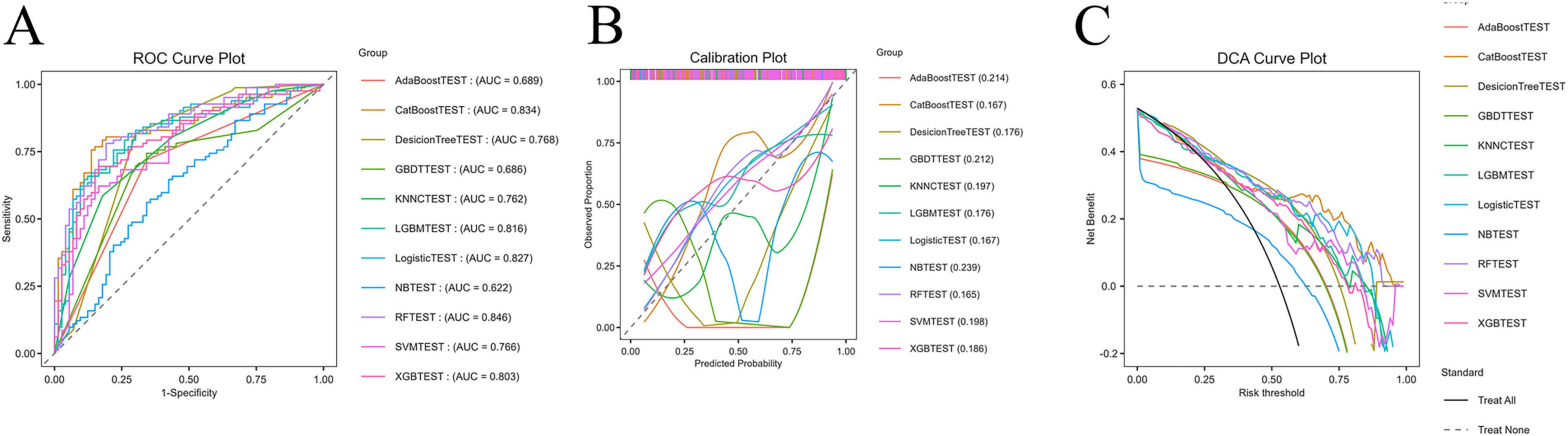
Performance evaluation of machine learning models for predicting cognitive impairment after Parkinson’s disease. **(A)** ROC curve plot showing the discriminative ability of different machine learning models in the test cohort. The Random Forest (RF) model achieved the highest predictive performance (AUC = 0.846), followed by CatBoost (AUC = 0.834) and Logistic Regression (AUC = 0.827), while AdaBoost and naïve bayes yielded relatively lower AUC values. **(B)** Calibration plots demonstrating the agreement between predicted probabilities and observed outcomes across models. The Random Forest, Logistic Regression, and Gradient Boosting Machine (GBM) models showed better calibration, indicating reliable probability estimation compared with other algorithms. **(C)** Decision curve analysis (DCA) illustrating the clinical utility of each model across a range of threshold probabilities. Most machine learning models, particularly Random Forest, Logistic Regression, and Support Vector Machine (SVM), provided greater net benefit than the “treat all” or “treat none” strategies, highlighting their potential clinical applicability.

As shown in Figure 2, receiver operating characteristic (ROC) analysis demonstrated that most ML models exhibited favorable discrimination for predicting cognitive impairment in PD. The RF model achieved the highest area under the curve (AUC: 0.846), followed closely by CatBoost (AUC: 0.834) and Logistic Regression (LR) (AUC: 0.827), whereas AdaBoost and Naïve Bayes performed less effectively. Calibration plots further indicated that RF, LR, and Gradient Boosting Machine maintained relatively good alignment between predicted and observed outcomes, suggesting stable probability estimation.

**Figure 3:**
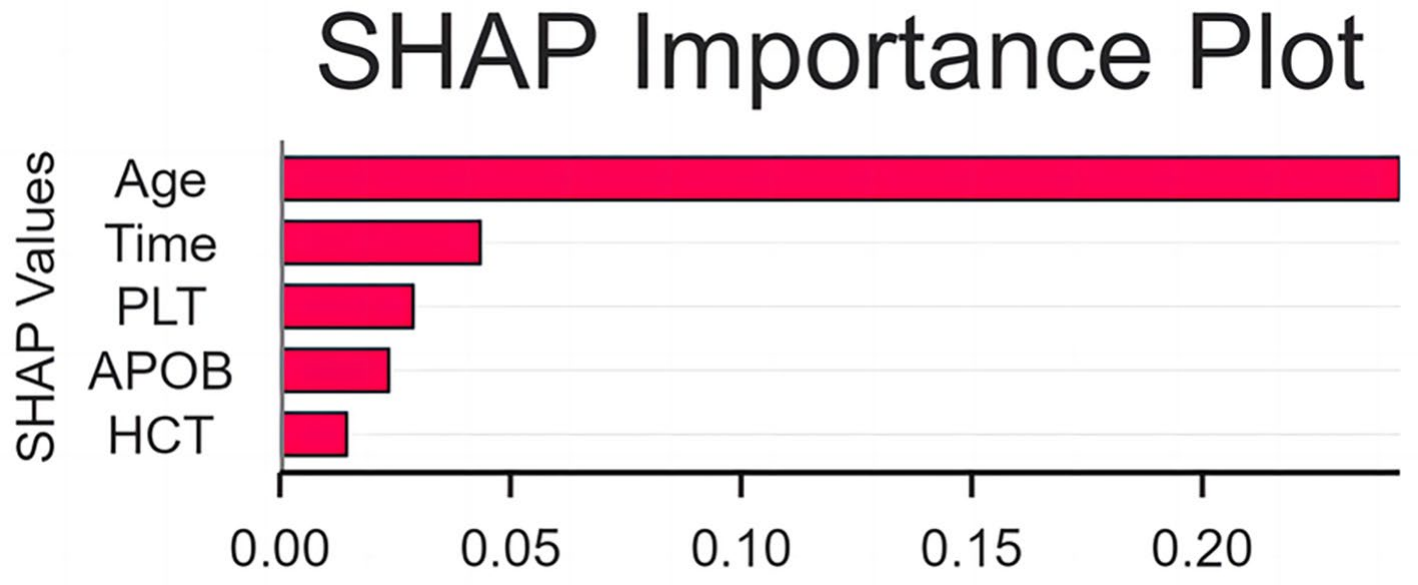
Global feature importance based on SHAP values. This bar plot depicts the mean absolute SHAP value for each of the top five predictive features in the final Random Forest model. The SHAP value represents the average magnitude of a feature’s contribution to the model’s output across all instances in the test cohort. The ranking confirms that age is the most influential predictor of cognitive impairment, followed by Time, between diagnosis and initial presentation to the hospital; PLT, platelet count; APOB, apolipoprotein B; HCT, hematocrit.

SHAP analyses were performed to improve the interpretability of the optimal RF model. The SHAP summary plot (Figure 3) demonstrates the effects of the top features for the model. It can be seen that the older Age and longer Time from diagnosis to presentation were the strongest predictors for higher risk of cognitive impairment. The higher values of APOB and HCT, on the other hand, were associated with the lower risk of CI, which was consistent with their protective effect. This individual-level explainability provides insights that the model’s decision is clinically intuitive.

### The model clinical utility

DCA revealed that several models, particularly RF, LR, and Support Vector Machine (SVM), consistently offered greater net clinical benefit across a wide range of threshold probabilities compared with “treat all” or “treat none” strategies. These findings underscore the clinical applicability of the optimized ML framework, with RF emerging as the most robust and reliable model (Figure 2).

### Performance metrics of the ML models

As presented in Table 3, most ML algorithms demonstrated satisfactory predictive performance, with mean scores of 0.709 for accuracy, 0.778 for recall, 0.737 for F1-score, and an average AUROC of 0.765. Among the models, RF achieved the best overall balance, with high AUROC (0.846), accuracy (0.75), and F1-score (0.775), indicating strong discriminative power and robustness. LR and CatBoost also performed well, yielding AUROCs of 0.827 and 0.834, respectively, coupled with recall values exceeding 0.80, suggesting reliable sensitivity for detecting cognitive impairment. Decision Tree and XGBoost provided acceptable results, though slightly lower than the top-performing models. In contrast, Naïve Bayes showed the weakest performance, with the lowest accuracy (0.59), F1-score (0.583), and MCC (0.196), reflecting limited predictive value. Overall, RF, LR, and CatBoost consistently emerged as the most reliable algorithms for cognitive impairment prediction, outperforming other models across multiple evaluation metrics (Table 3).

**Table 3:** Performance metrics of the machine learning model.

Model name	Accuracy	Prevalence	Recall	F1-score	MCC	AUROC	Presicion	Specificity	FNR	FPR
DesicionTreeTEST	0.76	0.529	0.817	0.784	0.521	0.768	0.753	0.700	0.183	0.301
GBDTTEST	0.69	0.529	0.720	0.711	0.378	0.686	0.702	0.658	0.280	0.342
AdaBoostTEST	0.69	0.529	0.720	0.711	0.378	0.688	0.702	0.658	0.280	0.342
LGBMTEST	0.74	0.529	0.829	0.768	0.471	0.816	0.716	0.630	0.171	0.370
LogisticTEST	0.74	0.529	0.854	0.773	0.474	0.827	0.707	0.603	0.146	0.397
RFTEST	0.75	0.529	0.817	0.775	0.495	0.846	0.736	0.671	0.183	0.329
NBTEST	0.59	0.529	0.537	0.583	0.1957	0.622	0.638	0.658	0.463	0.342
CatBoostTEST	0.74	0.529	0.817	0.766	0.470	0.834	0.720	0.643	0.183	0.356
XGBTEST	0.73	0.529	0.780	0.753	0.455	0.803	0.727	0.671	0.222	0.329
SVMTEST	0.69	0.529	0.866	0.747	0.390	0.766	0.657	0.493	0.134	0.507
KNNCTEST	0.69	0.529	0.805	0.733	0.379	0.762	0.673	0.561	0.195	0.438
Mean_scores	0.709	0.529	0.778	0.737	0.419	0.765	0.703	0.631	0.222	0.369

DecisionTreeTEST, Decision Tree Test; GBDTTEST, Gradient Boosting Decision Tree Test; AdaBoostTEST, Adaptive Boosting Test; LGBMTEST, Light Gradient Boosting Machine Test; LogisticTEST, Logistic Regression Test; RFTEST, Random Forest Test; NBTEST, Naïve Bayes Test; CatBoostTEST, Categorical Boosting Test; XGBTEST, eXtreme Gradient Boosting Test; SVMTEST, Support Vector Machine Test; KNNCTEST, K-Nearest Neighbors Classification Test; MCC, Matthews correlation coefficient; AUROC, Area under the receiver operating characteristic curve; FNR, False negative rate; FPR, False positive rate.

### Performance of RF models

**Figure 4:**
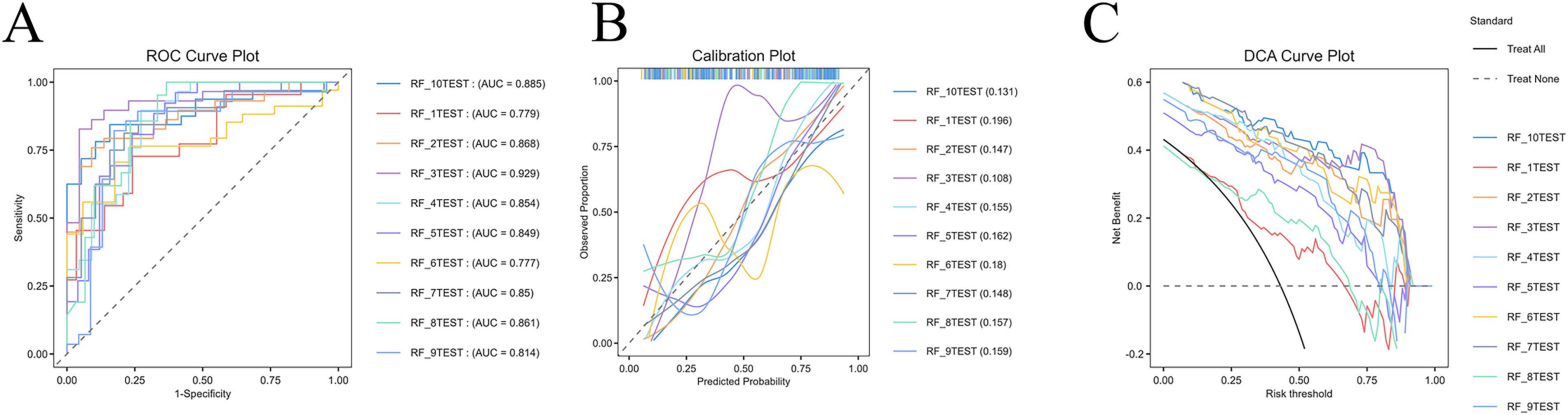
Performance evaluation of Random Forest models in predicting cognitive impairment after Parkinson’s disease. **(A)** ROC curves of ten Random Forest models in the test cohort. The majority of models achieved strong discriminative ability, with AUC values ranging from 0.779 to 0.929, and the RF_3 model showing the best overall performance (AUC = 0.929). **(B)** Calibration plots illustrating the agreement between predicted and observed probabilities. Several Random Forest models demonstrated good calibration, with curves aligning closely to the ideal diagonal, indicating reliable probability estimation across different thresholds. **(C)** DCA showing the net clinical benefit of the Random Forest models. Most variants consistently outperformed both the “treat all” and “treat none” strategies, demonstrating meaningful clinical utility across a wide range of risk thresholds.

As shown in Figure 4, the RF algorithm demonstrated consistently strong predictive ability across ten iterations in the test cohort. The area under the ROC curve (AUC) values ranged from 0.779 to 0.929, with the RF_3 model achieving the highest discriminative power (AUC = 0.929). These results highlight both the robustness and stability of RF in identifying patients at risk of cognitive impairment. Calibration analysis revealed that several RF models showed close alignment between predicted and observed probabilities, supporting the reliability of probability estimation. DCA further confirmed the clinical usefulness of these models, as most RF variants provided greater net benefit compared with “treat all” or “treat none” strategies across a wide range of threshold probabilities. Collectively, these findings establish RF as the most effective model for cognitive impairment prediction in this study.

### Validated model by the MoCA score using LR

**Figure 5:**
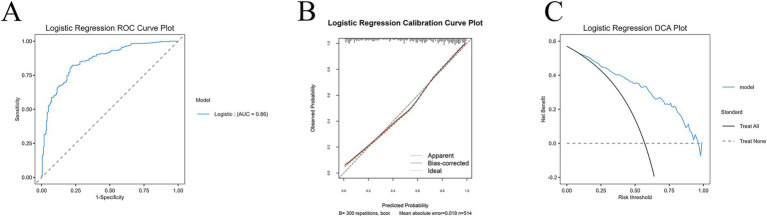
Logistic Regression model performance in predicting CI after PD by the MoCA score. **(A)** ROC curve showing the discriminative performance of the Logistic Regression model in the test cohort, with an AUC of 0.86, indicating strong predictive accuracy. **(B)** Calibration plot demonstrating close agreement between predicted probabilities and observed outcomes. The bias-corrected curve aligned well with the ideal diagonal, and the mean absolute error was low (0.019), supporting the reliability of the model’s probability estimates. **(C)** DCA illustrating the clinical utility of the Logistic Regression model. Across a wide range of threshold probabilities, the model consistently provided greater net benefit compared with both “treat all” and “treat none” strategies, highlighting its potential value for guiding clinical decision-making.

As illustrated in Figure 5, the Logistic Regression model demonstrated strong discriminative ability for predicting cognitive impairment in Parkinson’s disease. The ROC curve (Panel A) showed an area under the curve (AUC) of 0.86, indicating reliable classification performance between patients with and without cognitive impairment. Calibration analysis (Panel B) revealed close agreement between predicted and observed probabilities, with the bias-corrected line closely aligned with the ideal diagonal, and a mean absolute error of only 0.019 across 300 bootstrap resampling’s. These findings suggest that the model provided accurate probability estimation and was not overfitted. Furthermore, decision curve analysis (Panel C) confirmed the clinical utility of the Logistic Regression model. Across a wide range of threshold probabilities, the model consistently offered greater net clinical benefit compared to “treat all” or “treat none” strategies. Collectively, these results demonstrate that Logistic Regression is a robust and interpretable model with both high predictive accuracy and practical applicability for risk stratification in PD.

## Discussion

The current analysis developed and validated multiple ML models to predict CI in newly diagnosed patients with PD using baseline clinical and neuropsychological data. Among the algorithms tested, the RF model demonstrated the highest overall performance, achieving an AUROC of 0.846, with robust accuracy and F1-score across both training and test cohorts. Key predictive features included Age, PLT, Time from diagnosis to baseline visit, APOB, and HCT, suggesting that both demographic and routine laboratory variables carry significant prognostic value. The model’s reliability was further supported by calibration and decision curve analyses, which confirmed its stable probability estimates and clinical usefulness. These findings indicate that ML approaches—particularly tree-based models like RF—can effectively integrate heterogeneous outpatient data to enable early identification of PD patients at risk for CI, thereby offering a valuable tool for individualized care planning. The superior performance of RF may be attributed to its ability to handle non-linear relationships and interactions among variables without overfitting, which is particularly suitable for heterogeneous clinical datasets.

To provide more transparency of our best-performing RF model, the present research performed SHAP analysis and ‘opened the black box’ of the model. The resulting plots, displayed in Figure 3, offer global as well as individual level explainability. They allow for not only assessing the overall importance of the predicted model features and their direction (i.e., age and disease duration are positive risk factors), but also for importantly seeing how individual values of APOB and HCT drive the prediction toward or away from a CI diagnosis on an individual patient level. For example, one can see that a low HCT was the main driver for a high risk prediction for a certain patient. By thus moving away from simply providing accurate predictions to also offering explanations of these predictions.

The findings align with and extend previous research demonstrating that clinical and neuropsychological features are strong predictors of cognitive decline in PD. Similar to Schrag et al. who identified age, APOE status, and non-motor symptoms such as hyposmia and REM sleep behavior disorder as predictors of CI using a multimodal approach, the RF model also highlighted age and APOB as important features, supporting the role of both demographic and lipid-related factors in early cognitive prognosis (11). Unlike that study, however, the current analysis did not incorporate CSF or imaging biomarkers, and instead demonstrated that widely available clinical and laboratory data can achieve comparable predictive accuracy. The results also resonate with Savica et al. who found that gait parameters such as stride length and gait variability were associated with domain-specific cognitive decline; although the research did not explicitly include gait variables, the overlap in clinical markers like motor assessment scores suggests converging evidence on physical function as a cognitive marker (12). In contrast to studies like Pirogovsky-Turk et al. and Hu et al. which relied heavily on neuroimaging or task-specific neuropsychological assessments for prediction, our model emphasizes the value of a multi-domain, non-invasive approach using routine data to achieve clinically meaningful prediction in early PD cohorts (13, 14). This underscores a key contribution of the work: demonstrating that clinically accessible variables, when analyzed through ML frameworks, can rival more resource-intensive biomarker strategies for early risk stratification.

Multiple risk factors—including advanced age, longer disease duration, reduced HCT, APOB, and abnormal PLT activity—converge on several pathophysiological pathways to drive cognitive decline in PD. A key unifying mechanism is chronic neuroinflammation, wherein microglial activation and pro-inflammatory cytokine release are promoted by APOB-driven atherosclerosis, PLT-derived mediators, age-related immunosenescence, and hypoxia secondary to low HCT (15–18). Concurrently, these factors exacerbate cerebrovascular dysfunction and blood–brain barrier (BBB) disruption. APOB and platelet hyperactivity contribute to microvascular damage and thrombotic risk, while anemia (low HCT) reduces cerebral oxygen delivery, impairing neuronal metabolism (19). Age and disease duration further weaken vascular integrity. A compromised BBB facilitates the entry of peripheral inflammatory agents and impedes the clearance of toxic proteins. Additionally, mitochondrial dysfunction and oxidative stress are amplified by impaired oxygen utilization (HCT), metabolic dysregulation (APOB), and inflammatory signals. Finally, these processes create a permissive environment for pathological protein accumulation and spread—particularly *α*-synuclein aggregation—which propagates through vulnerable neural networks, ultimately leading to synaptic failure and cognitive impairment (20). Thus, these risk factors collectively accelerate PD-related cognitive decline by interacting through neuroinflammatory, vascular, metabolic, and proteinopathic mechanisms. Targeting these shared pathways may offer novel therapeutic strategies to preserve cognitive function in PD.

To further validate the robustness of the RF model, the stdy incorporated cognitive outcomes based on the MoCA in addition to the MMSE. While MMSE is widely used in clinical settings, it may lack sensitivity to detect early executive and visuospatial deficits common in PD. MoCA, on the other hand, is more sensitive to mild CI. By leveraging both tools, the RF model benefits from complementary diagnostic strengths, enabling more accurate identification of CI across different clinical profiles in PD patients.

### Limitation

This study has several limitations. First, while the sample size in this study was large enough to create the initial model, the study did not have external validation on a multi-center or prospective cohort. This could lead to limited generalizability to larger and more diverse populations of PD patients. External validation on an independent, multi-center dataset should be a future direction. Second, although our model demonstrated strong performance using clinical and neuropsychological features, the current analysis did not include imaging or CSF biomarkers, which may further enhance predictive accuracy. Third, the use of MMSE and MoCA as outcome measures, while clinically relevant, may be subject to educational and cultural bias. Lastly, the retrospective design may introduce unmeasured confounders, and external validation in prospective, multicenter cohorts is still needed. Future studies integrating multimodal biomarkers and real-world follow-up data will be critical to further improving the model’s clinical utility and robustness. Fourth, the 6-month follow-up duration is a limitation as it is relatively short for outcome prediction models. The natural history of cognitive decline in PD is protracted, with changes in cognition often occurring over several years. The purpose of this study was to identify early signals of risk and to enable earlier intervention, for which a 6-month time horizon is reasonable and can capture clinically meaningful shifts, particularly among those with more rapid progression or greater vulnerability at baseline. However, it is certainly too short to represent the long-term trajectory of cognitive decline, or to accurately predict conversion to Parkinson’s disease dementia. On the other hand, all the predictors in the final model have well-documented associations as longer-term risk factors (especially age, disease duration), which provides some face validity that these would apply over longer timescales as well. The model’s predictive accuracy for CI at 3–5 years is currently unknown, and should be explicitly tested in future prospective work with longer follow-up. Finally, potential effects of variants with well-established associations with PD (e.g., GBA or LRRK2) on cognitive end points were not investigated, because genetic testing was not systematically performed. Genetic data in future studies could be used to enhance the accuracy of the model and might reveal genotype-specific patterns of prediction.

## Conclusion

This study demonstrates that ML models using clinical and neuropsychological data can effectively predict early cognitive impairment in Parkinson’s disease. The Random Forest model showed strong performance, and validation with both MMSE and MoCA enhanced robustness. Th findings demonstrate that data-driven approaches can enhance early identification of cognitive decline in PD and support personalized clinical decision-making. Future research should focus on prospective validation and the integration of imaging and molecular biomarkers to further improve predictive precision.

## Data Availability

The raw data supporting the conclusions of this article will be made available by the authors, without undue reservation.
