# Supplementary material for: Predicting cognitive impairment in Parkinson’s disease: a machine learning approach based on clinical and neuropsychological data
Source: Front Neurol. 2025 Dec 19;16:1709386. doi: 10.3389/fneur.2025.1709386 (PMC12757295; doi:10.3389/fneur.2025.1709386)
Supplement: Supplementary file 1 [file Presentation_1.pdf]

Supplement: Literature review

PD analytics has been very recently expanded through deep temporal models (e.g., convolutional/pooling and LSTM units) by also leveraging metaheuristic optimization and gait sensing via wearable devices. A line of related work has in fact incorporated shoe-mounted accelerometer/gyroscope data streams into convolutional pipelines, employing modified metaheuristics (e.g., adaptive variants) to optimize the hyperparameters of models (CNN-based in most cases) for non-invasive PD recognition and yielding high agreement in multi-dataset test evaluations(1). Similarly, LSTM-based time-series classifiers for PD diagnosis with PD benchmark gait datasets —optimized with modified particle swarm or other metaheuristics—have also yielded improved accuracy/kappa scores(2–5) and have further provided an important window for model interpretability via SHAP feature analysis. Collectively, these sensor-driven and metaheuristic-tuned deep architectures inform a promising avenue for motor-phenotype recognition and freezing-of-gait detection specifically in PD. In comparison, our present study is more directly focused on a different (though also clinically urgent) predictive question — specifically, prospective prediction of cognitive impairment in PD—and takes a very different approach by restricting the input variables to those that are part of the routine clinical and neuropsychological battery (in an effort to maximize feasibility and scalability in outpatient care). This is a complementary approach to the prior literature, which seeks to demonstrate that strong, interpretable performance can be achieved without the need for specialized hardware.

Predicting the development of cognitive impairment (CI) in Parkinson's disease (PD) has been an ongoing subject of research, yet a viable predictive algorithm has not been created nor integrated in clinical practice. Epidemiological and longitudinal studies have solidified the high prevalence and progressive character of CI in PD and associated several risk factors including advanced age, disease severity, and the MAPT H1/H1 haplotype as some of the most predictive(6,7). The significant effect that CI and associated neuropsychiatric symptoms have on patient quality of life and caregiver burden has only further motivated the need to accurately predict its occurrence(8,9).

A great deal of effort has gone into identifying biomarkers of cognitive decline. For instance, several studies have pointed to uric acid as a strong biomarker of CI(10), with one of them also reporting a possible role of cystatin C(11). However, these results have been difficult to replicate, or have not seen wide use in clinical practice. Another area of interest has been in specific neuropsychological scales. Highly specialized tests such as the Mini Mental Parkinson scale or SCOPA-COG are likely great in following progression, but lack generalizability and sensitivity for a more widespread, early prognostic use(12). Other studies have implemented multiple scales, demographics, and other risk factors into a multimodal approach. Schrag et al. successfully created an algorithm to predict conversion to CI using a wide range of data, including age, APOE status, neuropsychiatric symptoms, and CSF biomarkers, and their model was able to show good performance(12,13). Other studies have found strong associations between specific domains and cognitive decline, such as gait

parameters in Savica et al.'s study(14), or baseline neuropsychiatric symptoms and certain neuropsychological deficits in Pirogovsky-Turk et al.'s work(15) and similar studies. A major limitation of many of these approaches is their emphasis on a data that are either only available from research (e.g. neuroimaging(16), CSF biomarkers [8], specific domain-restricted neuropsychological tests(15)), which, while potentially valuable in contributing to pathophysiological understanding, are extremely heterogeneous in outpatient clinics and difficult to access for the average patient in the real world. For a model to be used effectively and at scale, it has to be compatible with the tools at hand for the frontline clinicians. Our study aims to build on the previous work and implement machine learning approaches using only routine clinical and neuropsychological variables. This differentiates our model from others as it is the only one that can be directly applied to the real-world outpatient settings where the great majority of PD patients are treated, and has potential to scale well across different hospitals.

References

1. Jovanovic L, Damaševičius R, Matic R, Kabiljo M, Simic V, Kunjadic G, Antonijevic M, Zivkovic M, Bacanin N. Detecting parkinson's disease from shoe-mounted accelerometer sensors using convolutional neural networks optimized with modified metaheuristics. *PeerJ Comput Sci* (2024) 10:e2031. doi: 10.7717/peerj-cs.2031
2. Cuk A, Bezdan T, Jovanovic L, Antonijevic M, Stankovic M, Simic V, Zivkovic M, Bacanin N. Tuning attention based long-short term memory neural networks for parkinson's disease detection using modified metaheuristics. *Sci Rep* (2024) 14:4309. doi: 10.1038/s41598-024-54680-y
3. Bacanin N, Petrovic A, Jovanovic L, Zivkovic M, Zivkovic T, Sarac M. Parkinson's disease induced gain freezing detection using gated recurrent units optimized by modified crayfish optimization algorithm. *2024 5th International Conference on Mobile Computing and Sustainable Informatics (ICMCSI)*. Lalitpur,

Nepal: IEEE (2024). p. 1–8 doi: 10.1109/ICMCSI61536.2024.00006
4. Zivkovic M, Bacanin N, Zivkovic T, Jovanovic L, Kaljevic J, Antonijevic M. "Parkinson's detection from gait time series classification using LSTM tuned by modified RSA algorithm,." In: Kumar S, Hiranwal S, Garg R, Purohit SD, editors. *Proceedings of International Conference on Communication and Computational Technologies*. Lecture Notes in Networks and Systems. Singapore: Springer Nature Singapore (2024). p. 119–134 doi: 10.1007/978-981-97-7423-4_10
5. Markovic F, Jovanovic L, Spalevic P, Kaljevic J, Zivkovic M, Simic V, Shaker H, Bacanin N. Parkinsons detection from gait time series classification using modified metaheuristic optimized long short term memory. *Neural Process Lett* (2025) 57:14. doi: 10.1007/s11063-025-11735-z
6. Riedel O, Klotsche J, Spottke A, Deuschl G, Förstl H, Henn F, Heuser I, Oertel W, Reichmann H, Riederer P, et al. Cognitive impairment in 873 patients with idiopathic parkinson's disease: results from the german study on epidemiology of parkinson's disease with dementia (GEPAD). *J Neurol* (2008) 255:255–264. doi: 10.1007/s00415-008-0720-2
7. Williams-Gray CH, Evans JR, Goris A, Foltynie T, Ban M, Robbins TW, Brayne C, Kolachana BS, Weinberger DR, Sawcer SJ, et al. The distinct cognitive syndromes of parkinson's disease: 5 year follow-up of the CamPaIGN cohort. *Brain* (2009) 132:2958–2969. doi: 10.1093/brain/awp245
8. Juneja A, Anand K, Chandra M, Deshpande S, Dhamija R, Kathuria P, Mahajan R. Neuropsychiatric symptoms and caregiver burden in parkinson's disease. *Ann Indian Acad Neurol* (2020) 23:656. doi: 10.4103/aian.AIAN_91_20
9. Visser M, Verbaan D, Van Rooden S, Marinus J, Van Hilten J, Stiggelbout A. A longitudinal evaluation of health-related quality of life of patients with parkinson's disease. *Value Health* (2009) 12:392–396. doi: 10.1111/j.1524-4733.2008.00430.x
10. Annanmaki T, Pohja M, Parviainen T, Hakkinen P, Murros K. Uric acid and cognition in parkinson's disease: a follow-up study. *Park Relat Disord* (2011) 17:333–337. doi: 10.1016/j.parkreldis.2011.01.013
11. Hu W-D, Chen J, Mao C-J, Feng P, Yang Y-P, Luo W-F, Liu C-F. Elevated cystatin C levels are associated with cognitive impairment and progression of parkinson disease. *Cogn Behav Neurol* (2016) 29:144–149. doi: 10.1097/WNN.0000000000000100
12. Serrano-Dueñas M, Calero B, Serrano S, Serrano M, Coronel P. Metric properties of the mini-mental parkinson and SCOPA-COG scales for rating cognitive deterioration in parkinson's disease. *Mov Disord* (2010) 25:2555–2562. doi: 10.1002/mds.23322
13. Schrag A, Siddiqui UF, Anastasiou Z, Weintraub D, Schott JM. Clinical variables and biomarkers in prediction of cognitive impairment in patients with newly diagnosed parkinson's disease: a cohort study. *Lancet Neurol* (2017) 16:66–75. doi: 10.1016/S1474-4422(16)30328-3
14. Savica R, Wennberg AMV, Hagen C, Edwards K, Roberts RO, Hollman JH, Knopman DS, Boeve BF, Machulda MM, Petersen RC, et al. Comparison of gait parameters for predicting cognitive decline: the mayo clinic study of aging. *J*

Alzheimers Dis (2016) 55:559–567. doi: 10.3233/JAD-160697
15. Pirogovsky-Turk E, Moore RC, Filoteo JV, Litvan I, Song DD, Lessig SL, Schiehser DM. Neuropsychiatric predictors of cognitive decline in parkinson disease: a longitudinal study. *Am J Geriatr Psychiatry* (2017) 25:279–289. doi: 10.1016/j.jagp.2016.10.004
16. Ghadery C, Koshimori Y, Christopher L, Kim J, Rusjan P, Lang AE, Houle S, Strafella AP. The interaction between neuroinflammation and β-amyloid in cognitive decline in parkinson's disease. *Mol Neurobiol* (2020) 57:492–501. doi: 10.1007/s12035-019-01714-6
